# BAKE: a novel framework for iterative security design for identifying criminally-exploitable vulnerabilities in biotechnology products

**DOI:** 10.1186/s40163-025-00256-8

**Published:** 2025-10-02

**Authors:** Mariam Elgabry, Darren Nesbeth, Paul Ekblom, Shane Johnson

**Affiliations:** 1 DAWES Center for Future Crime at UCL, Jill Dando Institute for Security and Crime Science, 35 Tavistock Square, London, WC1H 9EZ UK; 2 UCL Biochemical Engineering, Bernard Katz, London, WC1E 6BT UK; 3 35 Tavistock Square, London, England, WC1H 9EZ UK; 4 Design Against Crime, University of the Arts London, London, UK

**Keywords:** Delphi, Hackathon, Governance, Medical device, Policy, Framework, Secure, Design

## Abstract

Emerging “in-body” monitoring, such as via ingestible devices, promises the future of personalised health, yet discussions of crime and security implications remain of low priority. Here, we develop and deploy the scenario building of the Delphi process and the prototyping of the hackathon through a hybrid hackathon Delphi framework that we have labelled “BAKE”. The aim of BAKE is to capture insight from experts regarding the risks posed by these devices; and to produce evidence for the utility of the model as a mechanism to identify at an early stage of design/development, criminally-exploitable vulnerabilities in biotechnology (bio-electronic devices), especially medical products/services. Findings from four expert groups include the identification of four crime forms (e.g., corporate exploitation, data breaches). Five secure by design principles (e.g., end-to-end encryption) and four governance mechanisms (e.g., independent body) were recognised. Four stakeholders were identified (e.g., technical, advocates for equitable treatment). Results indicate that the inclusion of non-traditional experts and early career researchers within the hackathon model can allow the identification of highly challenging threats within the cyber-physical device system. We demonstrated that hosting a hackathon with an embedded Delphi process can instigate secure by design thinking earlier in the product development life cycle of any emerging technology.

## Introduction

Security implications of new technology remain difficult to predict and are critical to future studies (Minkkinen, 2019). New technology, including biotechnology, can lead to new crime opportunities as security is often overlooked. The commercialisation of the Internet-of-Things (IoT), for example, introduced an unprecedented convenience to consumers by connecting everyday appliances to the internet wirelessly and through the use of sensors to provide additional functionality (Maple, 2017). At the same time, IoT has increased the attack surface of crime (Omolara et al., 2022; Roe et al., 2022) through the illegal use of the network to which they are connected in the case of burglary, stalking, and sex crimes (X, & Author 2021). With Cisco reports stating that there are three times as many smart connected devices as there are people,^1^ discussions of their crime and security implications must be frontline. Connected wearable devices (Kim et al., 2019) are widespread, amongst other types of connected “things” (Srinivasan, et al., 2019). “In-body” biotelemetry devices (Kiourti & Nikita, 2017) are emerging to include “next generation” ingestible devices (Alsunaydih & Yuce, 2021) containing integrated sensors—the Internet-of-*Ingestible*-Things.

Being connected may be conducive to 1 day achieving personalised health (Magni et al., 2021) but security expertise is often siloed and guidance fragmented. This causes uncertainty in conformity for manufacturers, especially for start-ups and Small and Medium sized Enterprises (SMEs) (Kitchin & Dodge, 2019). Security risk assessments are treated as a mere compliance check and security design is not considered. Although several organizations such as the US Food and Drug Administration (FDA), the International Organization for Standardization (ISO) and EU Medical Device Coordination Group (MDCG) are contributing to the elaboration of general standards for medical devices, they are not specific to emerging ingestible technology and often lag behind it. While these may cover basic cyber security hygiene, they are not designed to forecast crime implications.

Taken together, there is a need for better futures techniques that can (1) assess risks appertaining to new products, over a timescale that fits with the relevant product development/marketing cycle; (2) inform the design/development process from an early stage to avoid unsatisfactory late/retrofit incorporation and facilitate creative optimisation of conflicting requirements; (3) mesh well with the regulatory/governance environment and with the requirements of wider range of stakeholders.

In the following section we briefly explain the Delphi process, a technique available for horizon scanning, and how it can be paired with the hackathon model. To address the above-mentioned points, we also describe in the following section the hybrid hackathon Delphi framework that we have developed and labelled BAKE.^2^

### The Delphi process

The Delphi process (Dalkey & Helmer, 1963; Linstone and Turoff 1975; Turoff, 1970) is a common forecasting technique originally developed by the RAND corporation (Dalkey, 1968) to determine the consensus of a group of experts on a given complex problem. Briefly, it is a prescriptive process (Belton et al., 2019) comprising a series of iterative surveys that are distributed to a pre-determined panel of experts, where the group responses are aggregated after each round to inform the next series of responses. With each subsequent round, the variation in responses is expected to decrease as the group converges to an agreement or “consensus” (Avella, 2016). Early responses typically generate a wide array of alternatives as participants provide varying viewpoints/expertise, that distils in the subsequent rounds (Fischer, 1978). One of the main benefits of the Delphi methodology is that it draws on expert opinion where empirical data may be limited or lacking and avoids “group think” (Rowe and Wright 1996).

The Delphi process is often used for the prediction of the occurrence of future events in several industries, such as healthcare (Chang et al., 2010; Keeney et al., 2006), technology (Alon et al., 2019; Merfeld et al., 2019) and finance (Kozak & Iefremova, 2014; Velez et al., 2020). To a lesser extent, the Delphi process can be useful in forecasting crime (Coutorie, 1995), and is often used as a policy making tool (Akartuna et al., 2022; Giannarou & Zervas, 2014). For example, a 2022 Delphi study (Author et al. 2022) forecast trends in crime facilitated by biotechnology to anticipate what these might look like in the next 5 years. This is hard to determine as the class of crime event may have not yet occurred or remains under-reported, unreported or unknown. The study interviewed “traditional” field experts, such as government officials, researchers, or industry professionals and “non-traditional” experts, such as “biohackers” (individuals who practise science outside institutional contexts). By having two groups, the parallel Delphi captured current beliefs and concerns from the broader community that serve as a baseline for future study and the establishment of public policy.

While the 2022 parallel Delphi study (Author et al. 2022) generated insights for a wider biotechnology threat and solution landscape to allow for targeted prevention strategies to be established, it was limited to a scenario-building exercise. We sought to complement the Delphi process with an experimental approach using ingestible devices (see Steiger et al., 2019 for a review) as an example of emerging technology. The aim was to forecast the direction and form of security required for them—ahead of time. We selected ingestible devices as an emerging and potentially impactful technology for future health and chronic clinical applications (Chong & Woo, 2021).

### BAKE: the hybrid hackathon Delphi

The BAKE framework was devised to entail three phases: scenario building; prototyping; and assessing wider policy implications of the technology of interest. As future technology consumers are expected to fully participate in crime-detection (Graham & Mehmood, 2014), the Delphi process is complemented with an experimental approach of the hackathon and its entailed prototyping phase (Tucker et al., 2018). “Hackathon” refers to a design sprint-like event bringing domain experts to collaborate intensively on a project (Tucker et al., 2018). Although relatively new, the hackathon model has been useful to various applications such as medical technology innovation (e.g., DePasse et al., 2014), as an educational tool (e.g., Wang et al. 2018), or to solve social issues and business objectives (Briscoe, 2014). At its core, it invites diverse and creative individuals, from outside traditional backgrounds, to collaborate with an emphasis on a problem-based approach (DePasse et al., 2014).

The combination of the Delphi and hackathon addresses the need for a mechanism of responsible research innovation to systematically consider security implications of a technology during its design phase, rather than retrospectively fitting solutions at the end of the product life cycle. We investigate this model and recruit an internal group of early career researchers/students in the medical technology space by hosting a hackathon and conducting a parallel Delphi with external groups of experts, namely, non-traditional, traditional (as per Author et al. 2022) and patients.

BAKE combines the Delphi process with the hackathon model (Halvari et al., 2019). Where the 2022 Delphi study (Author et al. 2022) was limited to scenario building, the hackathon model compliments the speculative process by eliciting practical suggestions through an active design process that can further highlight issues (e.g., for policymakers). This hybrid framework has not been used before in the context of biotechnology crime preventive design: it allows for the translation of the outcomes into practical suggestions through a policy brief on secure by design principles for ingestible technology. The aim of this study is two-fold:

1. To capture nuanced insight from experts regarding the risks posed by these devices; and
2. To produce evidence for the utility of the model as a mechanism to identify at an early stage of design/development, criminally-exploitable vulnerabilities in electronic devices, especially biotechnology/medical products/services, using ingestible devices as a case study.

In the following section, we will describe the methodological approach taken, including participant selection, participant recruitment through a hackathon model, the questionnaire used, and the survey approach.

## Methods

### BAKE participants, recruitment and selection

#### Participant selection overview

Table 1 provides a summary of all participants who took part in BAKE, including eligibility criteria and recruitment methods. Participants were drawn from four distinct groups: hackathon participants, traditional experts, non-traditional experts, and patients.

**Table 1:** Participant description, eligibility and recruitment methods

	Hackathon participants	Traditional experts	Non-traditional experts	Patients	Additional (and pooled) experts
Eligibility criteria	● Early career researcher/student in medical technology ● Age range: 18–34 years old	● A level of training, biotechnology-related or security-related experience (1 or more years expertise), ● Age range: 20–70 years old	● Their recognition within the community (media records that discuss their activity), ● Age range: 20–70 years old	● Part of a patient society, have suffered from inflammatory bowel disease (Crohns or Ulcerative colitis) ● Age range: 20–70 years old	● A level of training, biotechnology-related, cybersecurity, medical device, regulatory or security-related experience (1 or more years expertise), ● Age range: 20–70 years old
Identification	Hosting a hackathon	Previous Delphi study using stakeholder mapping (including government, industry and academia)	Slack channel “grind syndicate” informed from preceding fieldwork (Author and X 2021)	Patient societies (Girl with Guts and Hellenic Crohn’s society)	Delphi participants from Round 3, additional cyber-biosecurity experts (contacted from joint authorship in cyber biosecurity book), Biohacking village speakers in DEFCON29 (2021), UK national medical device catapults and regulators, hackathon judges/speakers
Recruitment	Email	Email	Email	Email	Email
Description	2 Females 7 Males 1 participant from Ireland, 1 from Cyprus and the rest of the 7 participants were from the United Kingdom 8 Medical technology students 1 Early career professional within the Pharmaceutical industry	1 Female 4 Males All participants were from the United Kingdom 1 Academic in Biotechnology 1 Industry expert in IoT vulnerability testing 3 UK National security (and related)	All 3 Males All participants are Biohackers from the United States 1 Electrical Engineering/Cyborg 1 Genetic Engineering/Community Laboratory Founder 1 Implants/Cyborg	4 Female 2 Males 3 participants from Greece, 1 from the United Kingdom and 2 from the United States All participants were diagnosed patients with Crohn’s disease	16 Females 29 Males Participants were mainly from the United Kingdom and the United States, and some from Israel, Austria 11 Delphi participants from Round 3, 34 additional experts from UK national security organisations/agencies, International cyber-biosecurity experts in academia and industry, US Health Policy, UK medical technology catapults, UK Security Researcher, UK Defence/security Think Tank, UK Medical device regulating authorities, UK consumer devices regulating authority, UK and Austrian Police
Initially contacted	9	9	4	2	53 (23 Delphi participants, 30 additional)
Round 1	9	5	3	6	0
Round 2 and 3	9	5	3	6	0

^a^https://www.enisa.europa.eu/topics/standards/certification

#### Hackathon participant recruitment and process

The BAKE hackathon was hosted by the Dawes Centre for Future Crime and participants were recruited through the Medical Technology Society at University College London (UCL). In the first stage, 26 early-career researchers and students applied via an online form. Applicants were asked to describe their relevant skills and submit an ingestible device design proposal. This proposal assessed five key criteria: the problem, solution, market readiness, team, and implementation plan. A cash prize for the winning team served as an incentive (distributed as three Amazon vouchers dividing a total of £1000).

Out of the 26 applicants, nine were selected based on their responses from the submitted proposal and assigned to three cross-disciplinary teams to promote diverse skill integration. Each team received ten one-hour talks from stakeholders across fields like cyber-biosecurity, medical devices, and future crime.

**Fig. 1:**
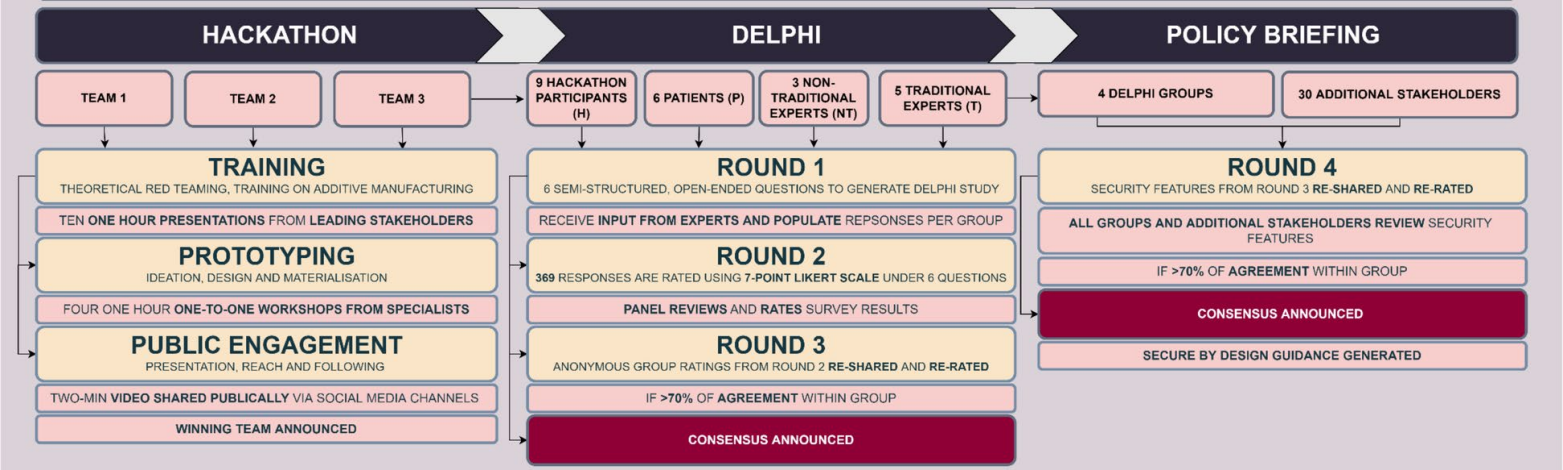
Overview of the Novel BAKE Framework. **Left Panel**: The first stage of the hackathon involved three finalist teams, each undergoing training, prototyping, and public engagement with their ingestible device designs. **Middle Panel**: The second stage presents the Delphi process, with the nine hackathon participants forming one group. Three additional expert groups participated in parallel: six patients, three non-traditional experts, and five traditional experts. The Delphi process comprised three rounds of the same six questions related to ingestible device technology (see Questionnaire in Methods). Each group generated responses independently, which were used to inform the second round. **Right Panel**: A fourth round focused on evaluating pooled security features identified across all groups. These were shared with additional external stakeholders, and consensus was defined as 70% or greater agreement across respondents. This final stage informed the development of a policy brief

After completing these training sessions, the three teams were tasked with designing a novel ingestible device to advance to the prototyping and public engagement phase (see Fig. 1). These three teams later contributed to the Delphi process. They were judged based on prototype creativity, security design, and presentation.

#### Delphi participant recruitment

Participants for the Delphi process were drawn from four groups:

- **Hackathon Participants (*n* = 9)**: As described above, these were selected from the three finalist teams.
- **Traditional Experts (*n* = 5)**: Recruited via networks within academia, industry, and UK government (e.g., UCL’s Institute of Health Engineering, departmental organizational charts for biotech-related agencies). Of the 9 contacted, 4 did not respond.
- **Non-Traditional Experts (*n* = 3)**: Identified through prior fieldwork and the “Grind Syndicate” Slack group (a community of biohackers). One of four initially interested individuals later withdrew.
- **Patients (*n* = 6)**: Recruited via patient organizations (e.g., the Hellenic Crohn’s Society and Girls with Guts). Administrators from these groups helped identify individuals living with Inflammatory Bowel Disease (IBD), a key target user group for ingestible devices.

#### Summary of Delphi participation

In total, 23 individuals participated in the Delphi study: 9 from the hackathon, 5 traditional experts, 3 non-traditional experts, and 6 patients. Each participant was provided with a study information sheet (Supplementary Fig. 1) and gave informed consent, with assurance of anonymity and the ability to withdraw at any time.

#### Note on delphi rounds and response rates

The Delphi study involved four rounds of feedback. Table 1 outlines the initial contact numbers and participation across each round. As the rounds progressed, some attrition occurred. A full explanation of the rounds is included later in the Methods section (see Sect. "Delphi procedure").

### BAKE study design

As shown in Fig. 1, the BAKE involved a 3-month process with three stages of: prototyping (hackathon), scenario building (Delphi) and assessing technology implications (policy briefing). The following sections describe the approach taken for each stage.

#### Internet-of-ingestible-things hackathon

The top three teams were selected to progress to the next stage (Fig. 1, Left Pa nel). These were selected based on a combination of i) public reach/following and ii) scoring (security design, prototype creativity and overall pitch showmanship) provided by a judging panel of industry experts in cybersecurity, medical devices and the IoT.

Teams received four one-hour one-to-one workshops (held virtually) from specialists in additive manufacturing (3D printing) and design, to prototype their ingestible devices. (Prototypes were produced at the Institute of Making, University College London.)

To engage with the public and raise awareness on ingestible device security, following the prototyping phase teams generated a two-minute video demonstrating their designs. These were shared through social media channels (Twitter, Facebook, Instagram etc.).

#### Delphi process

All selected team members from the hackathon were then invited to participate in a Delphi study (Dalkey & Helmer, 1963; Linstone and Turoff 1975; Turoff, 1970) that involved four survey rounds (Fig. 1 Middle Panel). An additional three groups (external to the hackathon) also underwent the Delphi process in parallel; t raditional, non-traditional experts, and a patient group (see Participants section below). Traditional and non-traditional expert groups, similar to the 2022 Delphi study conducted (Author et al. 2022), were included as comparison groups to the hackathon participants (who engaged with the issue more interactively than the just providing responses to the questionnaire). The patient group was added because of the additional insights that they could provide from a different (*user*) perspective (e.g., regarding the practical use of the ingestible device).

#### Delphi questionnaire

The survey consisted of six “open” questions (see below), intended to elicit as much information as possible and to identify “scenarios”.

1. What area(s) of the Ingestible Things might be misused for crime?
2. What forms of security should Ingestible Things have?
3. How should Ingestible Things be governed?
4. Who are Ingestible Things stakeholders?
5. What should be communicated to consumers of Ingestible Things?
6. Are there any other Ingestible Things concerns that we should be thinking about now?

The questionnaire was piloted by two beta-testers with expertise in cybersecurity and software development, and adjusted according to their feedback.

#### Delphi procedure

In the first survey round, participants in each group were individually asked the above questionnaire, either through a virtual interview or a digital survey questionnaire (sent via email), depending upon availability of the participant during the timeframe of the study.

In the first round, the responses generated by each group were analysed to identify themes (Thomas & Harden, 2008). These formed the material used in the second round. Each group was presented with the same six questions and the themes that emerged for their group, and asked to rate them according to the extent to which they agreed with them. Participants were also encouraged to comment at the end of the questionnaire.

A summary of the groups’ overall ratings was sent to them in the third round, using the electronic platform TypeForm.com. All participants were encouraged to comment at the end of the questionnaire and invited to consider changing their ratings (none did).

In contrast to the 2022 Delphi study conducted (Author et al. 2022), this Delphi comprised an additional round. In round four, a summary of the responses generated across all four groups regarding the security features (for the ingestible devices) were presented (Fig. 1). The questionnaire was sent to all participants of all groups via email using TypeForm and they were asked to rate the extent to which they agreed with them on a 7-point Likert scale (one indicating strong agreement, and seven strong disagreement). All participants were encouraged to comment at the end of the questionnaire. The survey was also sent to additional participants who did not participate in the first three rounds of the Delphi for further contribution (see Recruitment and Selection below).

#### Analysis of Delphi outputs

Consensus was defined for each item by scoring more than 70% in ‘Strongly Agree’ within each group, in line with commonly used definitions of consensus (Vogel et al., 2019; see Giannarou & Zervas, 2014 for a review). The resulting scenarios for which consensus was reached, were then grouped by topic.

## Results

The presentation of findings is divided into sections for each question asked relating to the identified crime forms (Sect. "Crime forms revealed by BAKE"), proposed security features (Sects. "Irresponsible manufacturer" and "Corporate espionage and exploitation") and consumer communications (Sect. "Data breaches and system vulnerabilities for sabotage, crime and extortion"), identified stakeholders (Sect. "Security focus areas derived from BAKE"), expected governance (Sect. "Secure by design principles from BAKE") and any other concerns about ingestible technology (Sect. "Consumer communication areas identified by BAKE"). For each of the six topics discussed, the responses from the (internal) hackathon group (*H*) are discussed first and then compared to the (external) groups of patients, traditional and non-traditional experts (*P*, *T*, NT). The results of the fourth round of the Delphi process are discussed within the identified security features section (and provided the basis of the policy briefing) (Sect. "Data breaches and system vulnerabilities for sabotage, crime and extortion").

**Fig. 2:**
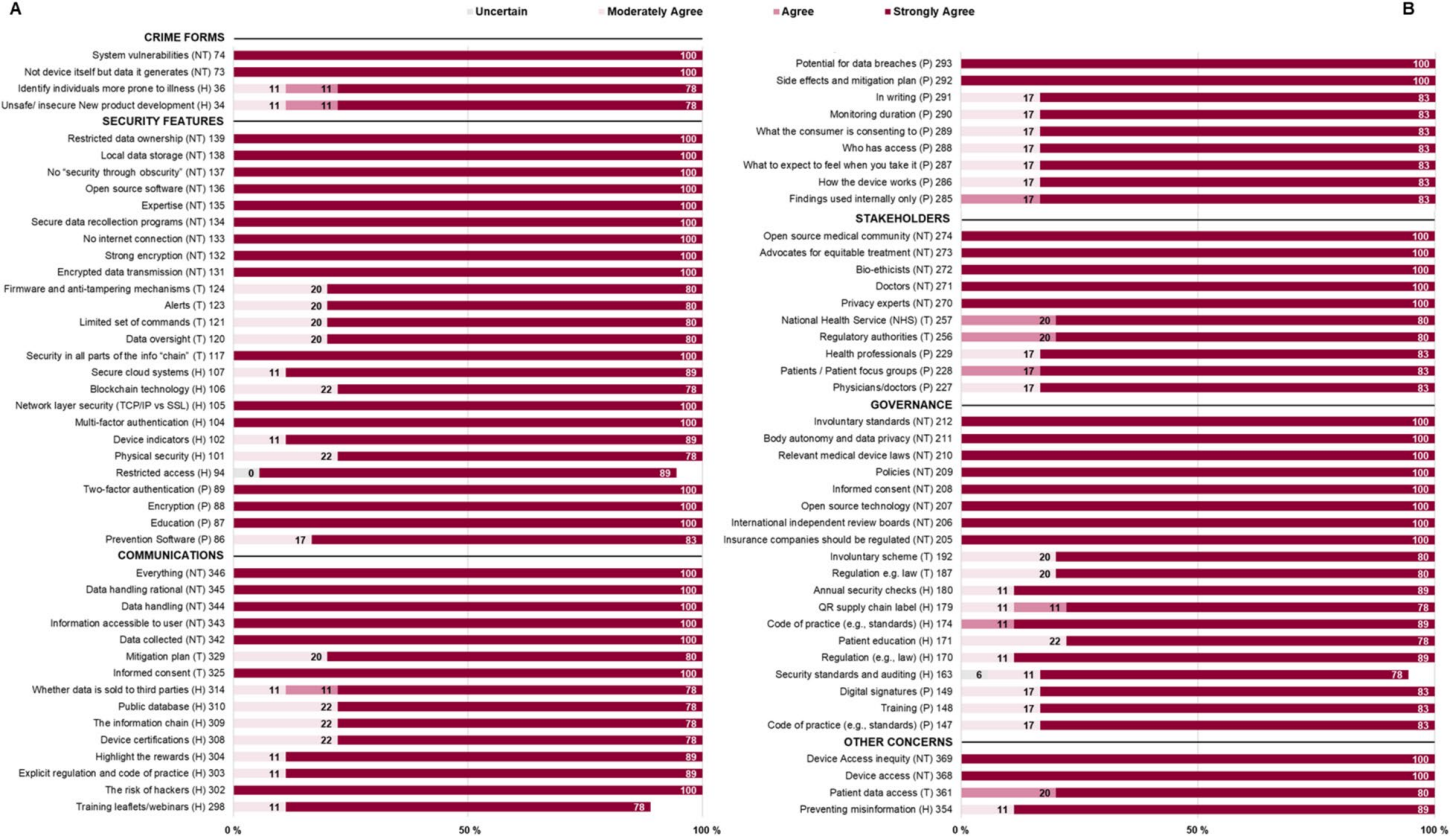
Consensus on the Internet of Ingestible Things. All six questions with consensus responses divided into two panels (A) and (B) across all expert groups. Hackathon participant group (H), Patient group (P), Traditional (T) and Non-Traditional (NT) groups. Items were then grouped by common theme to produce the summary schematic in Fig. 3

As shown in Table 2, some 369 unique suggestions were generated by the four groups across the six questions asked. Consensus (> 70% strongly agree) was reached for 86 of the scenarios: these are presented in this section, Table 3 and Fig. 2.

**Table 2:** Total scenario counts per expert group

Group	Crime Forms	Security Features	Communications	Stakeholders	Governance	OTHER CONCERNS	GRAND TOTAL
H	23	18	21	19	31	7	119
NT	18	15	16	16	18	8	91
P	13	15	19	19	10	2	78
T	20	17	16	8	14	6	81
Grand Total	74	65	72	62	73	23	369

Hackathon participant group (H), Patient group (P), Traditional (T) and Non-Traditional (NT) groups

**Table 3:** Consensus scenario counts per expert group

Group	Crime Forms	Security Features	Communications	Stakeholders	Governance	OTHER CONCERNS	GRAND TOTAL
H	2	7	8	0	6	1	24
NT	2	9	5	5	8	2	31
P	0	4	9	3	3	0	19
T	0	5	2	2	2	1	12
Grand Total	4	25	24	10	19	4	86

Hackathon participant group (H), Patient group (P), Traditional (T) and Non-Traditional (NT) groups

### Crime forms revealed by BAKE

Consensus was reached for four crime forms: two generated by the hackathon participant group and two by the non-traditional expert group. We summarise all responses with strong agreement, and for each issue list the groups associate with each response as bullet points.

### Irresponsible manufacturer

- Hackathon participant group
  Ingestible devices will be subject to overlooked security by manufacturers (Fig. 2 #34). They suggested that overlooking the significance of securing the device to achieve a faster go-to-market strategy should itself be considered criminal.

### Corporate espionage and exploitation

- Hackathon participant group
  Corporate exploitation could take the form of corporate/research company/organisations misusing ingestible devices for non-consensual research to identify individuals who are more prone to illness (Fig. 2 #36). According to these participants, this could be misused (say) for influencing consumer purchasing behaviour, or targeted marketing.

One participant noted, “*Exploitation of consumer behaviour—tracking/monitoring of information (e.g. bacteria levels) from ingestible device to influence purchasing behaviour (e.g. food/probiotic products)*” (H5), while another explained “*Targeted marketing will be a growing issue. For example, if confidential data were to be leaked from a capsule, then the data could be sold or manipulated to benefit a third party like a drug marketing agency*” (H8).

Traditional experts did not reach consensus.^3^

### Data breaches and system vulnerabilities for sabotage, crime and extortion

- Non-traditional experts
  Data breaches of ingestible devices are expected as an identified crime form Fig. 2 #73, with one non-traditional expert noting that “*Currently we see at least a few high profile data breaches each year. This brings up some concerns regarding smart health products*” (NT2). Additionally, that the system (e.g., the network the device is connected to, or human element prone to social engineering attacks) surrounding the ingestible device is always at risk, (Fig. 2, #74). The patient^4^ and traditional expert^5^ groups did not reach consensus.

**Strong Disagreement** 6 **over Three Crime Forms**

Non-traditional expert group

The first was that ingestible devices could be used to scam people (Supplementary Table 1 #60 NT). The second was that ingestible devices may enable the creation of a social credit system (Supplementary Table 1 #59 NT). Finally, the third was that ingestible devices cannot yet be misused due to the technical barrier of their currently short read range (Supplementary Table 1 #57 NT).

### Security focus areas derived from BAKE

Consensus was reached for a total of five security focus areas, within all four expert groups,. The fourth round of the Delphi process across all four groups and additional stakeholders resulted in consensus for five Secure by Design principles. These results are discussed below.

**Device**

- Hackathon participant group
  That the ingestible devices would require device indicators that display whether the device is still functioning properly (e.g., battery working) for consumption (Figure 2 #102).
- Non-traditional expert group
  That the device should not be connected to the internet at all (Figure 2 #133).

**User Interface and Mobile application**

- Hackathon participant group
- Traditional expert group
- Non-traditional expert group
- Patient group

That the user interface and/or mobile application for ingestible technology will require additional security features. Specifically, that multi-factor authentication will be required to grant access to the user securely (Fig. 2 #104). Similarly, that two-factor authentication for the mobile application is a necessary security feature (Fig. 2 #89). Additionally, that the software of ingestible technology should be programmed so data leaks are prevented to the best of the software’s capabilities (Fig. 2 #86) and that strong encryption is vital (Fig. 2 #132). Alerts and/or notifications would be useful mechanisms to inform users if the device and/or system has been maliciously accessed remotely (Fig. 2 #123). Moreover, that the devices should have a limited set of commands that can be remotely controlled from the mobile app and that these should be clearly documented (Fig. 2 #121).

**Data and Data Communications**

- Hackathon participant group
- Traditional expert group
- Non-traditional expert group
- Patient group

Data and data communication protocols need to be secured and that a network layer will be required to secure data transmission with encryption (Fig. 2 #105). Similarly, that the transmission between the ingestible device and the receiver should be encrypted (Fig. 2 #131). Moreover, that if the devices are ingested, spend significant amounts of time in the gut or collect significant data, then recollection programs should be rigorous, (Fig. 2 #134) and that data access should be restricted (Fig. 2 #139) between the provider and the patient, stored locally (as opposed to a Cloud service) (Fig. 2 #138), be encrypted (Fig. 2 #88) and kept confidential (#120).

**System Security Features**

- Hackathon participant group
- Traditional expert group
- Non-traditional expert group
- Patient group

That the system (e.g., network, hospital) into which the ingestible devices are incorporated will require attention and that secure cloud systems (Fig. 2 #107) with restricted access (Fig. 2 #94), physical security and a digital firewall to prevent any hacks or modifications on the ingestible device (Fig. 2 #101) will be necessary. Blockchain technology could be used (Fig. 2 #106) for authenticity and transparency purposes. For example, certification that traces and verifies the device’s path from production to the end user.

Open Source (non-proprietary) software (Fig. 2 #136) and “*No security through obscurity*” (Fig. 2 #137) should be considered for the ingestible device technology and that there is a requirement for formal standards of security in all parts of the information “chain”—the ingestible device, the transfer of data from the devices and the eventual place of data storage (Fig. 2 #117).

Physician and patient education will be key (Fig. 2 #87) in trusting, selecting and consuming the ingestible devices.

**Design Life Cycle (NT)**

- Non-traditional expert group
  A diverse set of expertise is needed earlier in the product development life cycle of ingestible devices, including bio-ethicists, privacy, and encryption experts (Fig. 2 #135).

### Secure by design principles from BAKE

**Fig. 3:**
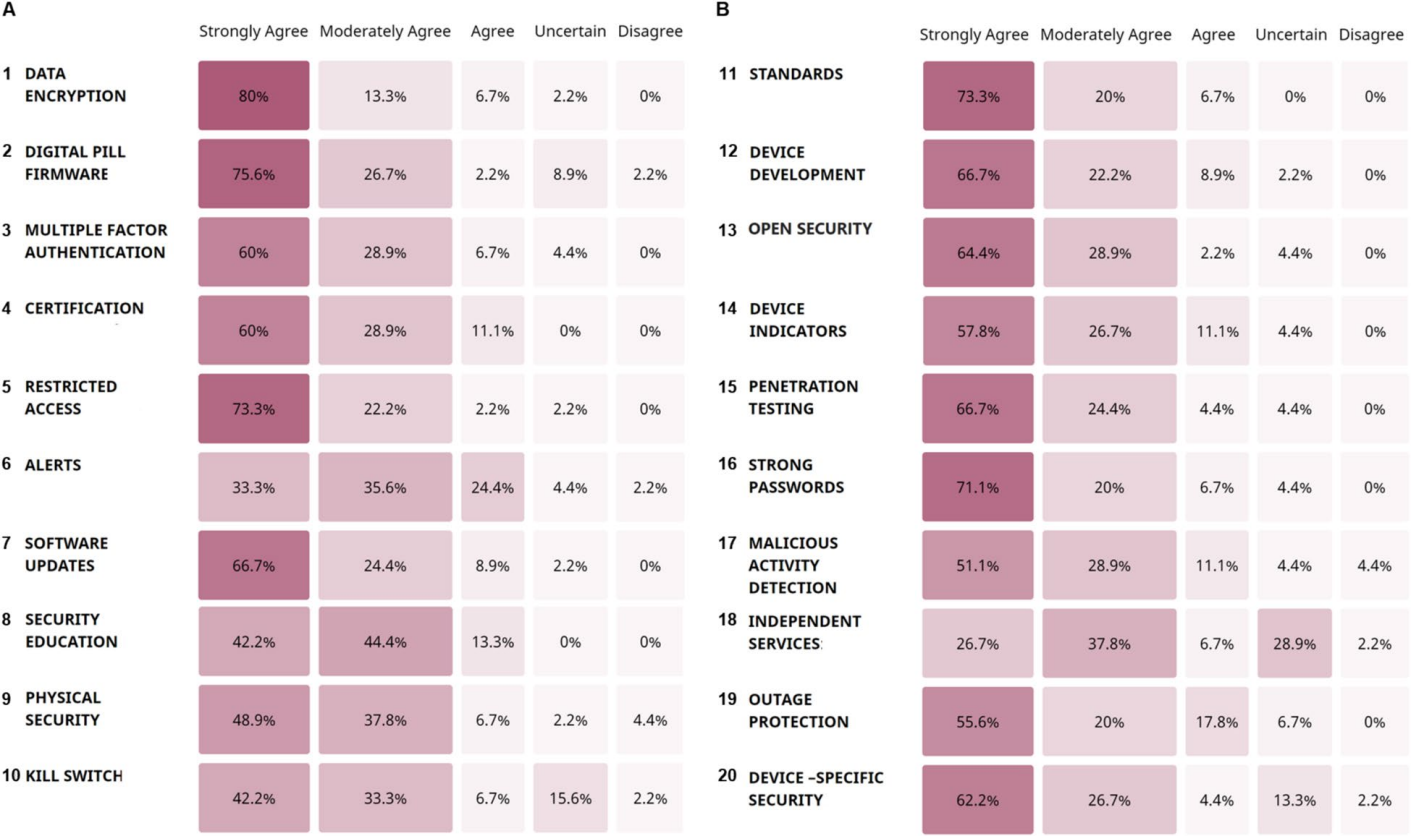
Secure by design principles with consensus agreement from the BAKE Framework. Responses indicated as a percentage from 45 responders, divided in two panels (A) and (B). Only top 5 of 7-point Likert scale shown—as the remaining was all zero%/unselected. The twenty items were ordered by placing the items for which all four groups strongly agreed (summative of ‘Agree’ and ‘Strongly Agree’ is greater than 50%). Following this, items that three groups strongly agreed with, then items that two groups strongly agreed with. For items that were unique to a single group, they were ordered with the highest percentage first

Figure 3 displays the responses of the fourth round of the Delphi process for reaching consensus on the security features expected by the participants for the ingestible devices (Question 2).

Consensus was reached for a total of five Secure by Design principles, across all four expert groups and additional stakeholders:

- Securing all data via end-to-end encryption (80%).
- Requiring device manufacturers to install up-to-date firmware (75.6%).
- Further restricting data and access to authorized personnel (73.3%).
- Ensuring that a formal set of standards (73.3%) of security are established/applied to all levels of the system in which the ingestible device is incorporated.
- Ensuring that strong passwords (71.1%) are established for the device and mobile application (i.e. no default passwords from manufacturers).

Seven principles with over 60% agreement but not reaching the 70% consensus threshold comprised (Fig. 3):

- Keeping software up-to-date (66.7%).
- Open-source (non-proprietary) (64.4%).
- Penetration testing (66.7%).
- Multiple factor authentication (60%).
- Device security certification by an independent authority (60%).
- Device-specific security features (62.2%).
- Involvement of diverse expertise (e.g., bio-ethicists, privacy experts) earlier in product development (66.7%).

### Consumer communication areas identified by BAKE

Consensus was reached within each expert group for a total of five areas that the expert groups suggested should be explicitly communicated to the consumer of ingestible technology.

**Functional Information**

- Hackathon participant group
- Non-traditional expert group
- Patient group

Functional information about the device should be clearly communicated to consumers and that ingestible device certifications to indicate security and safety could be useful (Fig. 2 #308). Information accessible to users should be conveyed (Fig. 2 #343), such as how the device works and what it does (Fig. 2 #286), what to expect to feel when ingesting the device (Fig. 2 #287) and the monitoring duration of the device (Fig. 2 #290).

**Security Information**

- Hackathon participant group
- Traditional expert group
- Non-traditional expert group
- Patient group

Security information should be communicated to consumers (Fig. 2 #346) for full transparency and informed consent so that they are aware of the risk of the ingestible device and/or system being exploited by malicious actors (Fig. 2 #302). At the same time, there should be explicit regulation and a code of practice explaining how ingestible device security is covered by data protection acts, say. Companies/manufacturers of ingestible technology need to be transparent about the potential of data breaches and explicit about what systems and mitigation plans are in place to prevent them (Fig. 2 #293, #329).

**Privacy Information**

- Hackathon participant group
- Non-traditional expert group
- Patient group

Information that the capsule collects should be clearly indicated, as well as who has access to that information, for how long, how it is handled, processed and stored (Fig. 2 #309H, #288P, #342NT, #344NT). Moreover, that users and consumers should be informed whether data is collected and sold to third parties (Fig. 2 #314). Similarly, that all data and findings received from the ingestible devices should be kept private and should only be used anonymously for internal use (Fig. 2 #285) and that the consumer should be made aware what it is that they are consenting to (Fig. 2 #289). The rationale behind most of the choices made in terms of data handling, storage and processing should also be explicitly communicated to users (Fig. 2 #345).

**Safety Information**

- Hackathon participant group
- Traditional expert group
- Patient group

Benefits and risks of the ingestible devices should be highlighted (Fig. 2 #304) to allow informed consent for their use (Fig. 2 #325). Similarly, that possible side effects, and what to do if one is experienced (where and how to report it), should be made available to users (including security issues) (Fig. 2 #292).

**Means of Communication**

- Hackathon participant group
- Patient group

Training leaflets and webinars to help patients/users to combat hackers/reduce any risks are appropriate means of communication (Fig. 2 #298). Additionally, that different pills may have different risks—and that these differences should be publicly available in a database containing all the types of ingestible devices with their features (Fig. 2 #310). All information should be presented at least in writing in simple language understandable to patients (Fig. 2 #291).

### Stakeholders defined by BAKE

Consensus was reached within each group except for the hackathon participants. Four types of stakeholders were identified for the ingestible technology.

**Health Professionals**

- Traditional expert group
- Patient group
- Non-traditional expert group

Health professionals are important stakeholders for ingestible technology, e.g., physicians, doctors, patients/patient focus groups (Fig. 2 #257, #227, #228, #229, #271).

**Information Technology Experts**

- Non-traditional expert group
  Privacy experts (Fig. 2 #270) and an open source medical community should play a large role in keeping the corporations and manufacturers honest. In doing so, source code made public can be checked and monitored to ensure that the ingestible device and related technology maintains security as they get checked by more individuals, more frequently (Fig. 2 #274).

Hackathon participants did not reach consensus.^7^

**Regulatory Authorities**

- Traditional expert group
  Major stakeholders comprise typical regulatory authorities e.g., Medicines and Healthcare products Regulatory Agency (MHRA), European Medicines Agency (EMA), Food and Drug Administration (FDA) (Fig. 2 #256).

Hackathon participants did not reach consensus.^8^

**Social Science Academics**

- Non-traditional expert group
  Bio-ethicists (Fig. 2 #272) and advocates for equitable treatment (Fig. 2 #273) should be vital stakeholders of ingestible technology.

**Strong Disagreement Regarding Stakeholders within Expert Groups**

- Non-traditional expert group
  Insurance companies are stakeholders for ingestible technology (Figure 2 #259).
- Traditional expert group
  A limited number of bodies/organisations should be providing oversight to the ecosystem/technology to avoid “mixed messaging” (Figure 2 #251).
- Patient group
  Patient families are stakeholders of ingestible devices (Figure 2 #213).

**Governance Mechanisms Defined by BAKE**

Consensus within all four expert groups was reached on four potential governance mechanisms that could be applied to the ingestible technology.

**Regulation**

- Hackathon participant group
- Traditional expert group
- Non-traditional expert group

Regulation through laws will be a required governance mechanism for the ingestible devices (Fig. 2 #170, #187, #210).

Specifically, that current medical device laws and regulations do not account for the rapidly developing biotechnology and needs concerning body autonomy and data privacy (Fig. 2 #211), with one non-traditional expert noting, ‘*Our systems of governance regarding medical devices, body autonomy and data privacy are severely behind the times and each and every facet of these would need to be updated to protect consumers in a world with embedded and ingested technology*.’ (NT3).

Moreover and in line with the strong disagreement indicated in the Stakeholder Section, insurance companies should be specifically targeted for regulation to safeguard against such privacy and security concerns (Fig. 2 #205).

**Standards and Code of Practice**

- Hackathon participant group
- Non-traditional expert group
- Patient group

Standards and code of practice for ingestible devices will be essential (Fig. 2 #147, #174, #209). Additionally, that annual quality assurance checks specifically for the updated security of ingestible devices should be enforced to ensure that manufacturers are keeping up with standards (Fig. 2 #180).

Standards and code of practice should be crafted in such a manner that they specifically cover the use of information generated by ingestible technology—and that this information is not used in any setting that is not explicitly consented to by the patient. This includes law enforcement, insurance, employment, and military uses (Fig. 2 #209).

**Transparent Technology and Education**

- Hackathon participant group
- Non-traditional expert group
- Patient group

Transparent governance mechanisms for ingestible devices are key and suggested that blockchain technology may be suitable (Fig. 2 #179). Specifically, and as an example, a QR code incorporated in the supply chain as a label to signify authenticity and transparent transactions for each device can be deployed and tracked through a public Blockchain. This could include certification that traces and verifies the device’s path from production to the end user—“smart contracts” (contractual digital arrangement given predetermined conditions are met (Buterin 2014) between providers for the different components of the ingestible device. In this manner, the final user can independently verify the quality of the received product. Digital signatures (Fig. 2 #149) as a governance mechanism of each individual device and its usage was suggested. Together,, users can have a better informed consent on the utility of ingestible technology (Fig. 2 #208).

A non-proprietary platform for the ingestible technology (o pen sourcing the code/platform) (Fig. 2 #207) will go much further than, say, corporate volunteerism and that education and training for patients (Fig. 2 #171) users, nurses, medical staff (Fig. 2 #148) will be necessary for successful (and responsible) governance of ingestible devices.

**Third Party/Independent Body**

- Non-traditional expert group
  International and independent review board is required to govern ingestible devices. This board, would be “air-gapped” financially to prevent any conflict of interest (Fig. 2 #206).

### BAKE consensus on misinformation and data access inequity

Consensus was reached for two additional concerns related to the ingestible technology from two expert groups.

**Misinformation**

- Hackathon participant group
  There will be a need to increase the trust on the part of consumers/users. Specifically, building trust so as to control the misinformation and conspiracy theories, seen with vaccines and other technology (Fig. 2 #354).

**Device/data Access/inequity**

- Non-traditional expert group
- Traditional expert group

Device and data access requires careful attention to prevent health inequity (Fig. 2 #368, 369).

Traditional experts did not reach consensus.^9^

## Discussion

Crime trends and security implications of technology remain difficult to predict. Yet advances in connected technology, as with other technology, continue to outpace those in their security. BAKE combined the scenario-building strengths of the Delphi process with the prototyping from the hackathon method to elicit forecasts from experts that help anticipate emerging trends while incorporating security within the design process. The validation of this holistic framework was pursued in support of responsible research innovation (Stilgoe et al., 2013) with practical implications for policy. While we cannot demonstrate that BAKE produces more or more useful information, interestingly the hackathon group of the BAKE Framework had no attrition during the Delphi study. Considering the high levels of attrition common to Delphi studies, the hackathon model seems to generate invested and committed participants.

In this section, we discuss the results and their implications. Firstly, we cover data security issues, and how the secure by design principles generated in this study relate to current regulation. Next, we discuss the crime forms identified in this study and how they connect to the challenges of cyber-physical technology (such as ingestible devices). Following this, we discuss the governance mechanisms suggested by study participants and the findings of the key stakeholders for ingestible technology given the unique challenges of cyber-biosecurity (Murch et al. 2018; Peccould et al. 2017). We conclude with the proposition of using BAKE as a framework and aid to help address some of these challenges.

### Expert view on data safeguarding via third party verification and unbiased design

Of the six questions investigated, and when comparing the topics generated, interestingly, only the security focus areas reached consensus within and across all four groups. The main concern was about data, supporting the seven principles of privacy by design, e.g., privacy by default, end-to-end security and privacy embedded into design (Cavoukian, 2009). Modern medical technology harnesses sensitive and personally identifiable data. Unfortunately, health data are increasingly becoming more valuable and a target for malicious actors (Yaqoob et al., 2019). The data ingestible devices generate includes genetic, lifestyle and environmental information about the user, besides their general health, making it even more identifiable (and desirable) than, for example, genetic material (Franzosa et al., 2015). Further research is needed on ensuring data is verifiably anonymous.

According to non-traditional experts, to verify anonymity data should be provided firstly to a trusted third party (Fig. 2 #206). This could be achieved by sampling the data transferred initially or at regular intervals to ensure standards are followed. Beyond data access by malicious hackers, the social harm manufactured into monitoring devices should be considered in the context of data sold to third parties. An example is the data leakage scandal whereby commercial DNA testing kit provider 23AndMe sold thousands of customers’ personal data to GlaxoSmithKline for $300 million (Brodwin, 2018) without customer consent. All four expert groups agreed that such data should be kept confidential and for internal use. In fact, recent regulatory proposals such as the UK’s Online Harms Act (2021) emphasize the “duty of care” of companies that include their products being subject to safety by design and GDPR regulations, violating such guidance would not constitute result a criminal offence. Hackathon participants, however, suggested that overlooking the significance of securing the device and the data generated, at the price of a faster go-to-market strategy, should itself constitute a crime (Fig. 2 #34).

All expert groups agreed that data processing should be fully disclosed to those who provide the data (Fig. 2 #13, #138, #120). Considering these findings, we suggest that manufacturers could benefit from additional consideration to data verification by users, especially if their ingestible technology involves using the data generated to train machine learning models to guide health decisions. An example could be if an ingestible product is used to guide interventions (e.g., extra care, pharmaceutical treatments, and lifestyle changes) based on the data of the user. This offers scope for political, race, gender, religious, social or other bias. Manufacturers must consider the design of the ingestible device to avoid a design that in physical operation or data handling may disadvantage certain demographic groups, generating health inequity (e.g., Obermeyer et al. 2019; Kadambi, 2021). In relation to ingestible technology adoption, other social implications such as misinformation (false information that may be honestly shared or well-intentioned) and disinformation (intentionally and maliciously produced and spread), as highlighted by H ackathon participants (Fig. 2 #354), also needs careful consideration. For example, just as conspiracy theories developed during the COVID19 pandemic (Enders et al., 2020; Pummerer et al., 2022), ingestible technology could be considered as a tracking device for unlawful covert surveillance—as identified by Hackathon participants and traditional experts (Supplementary Table 1 #39).

### Secure by design and current regulatory guidance

**Fig. 4:**
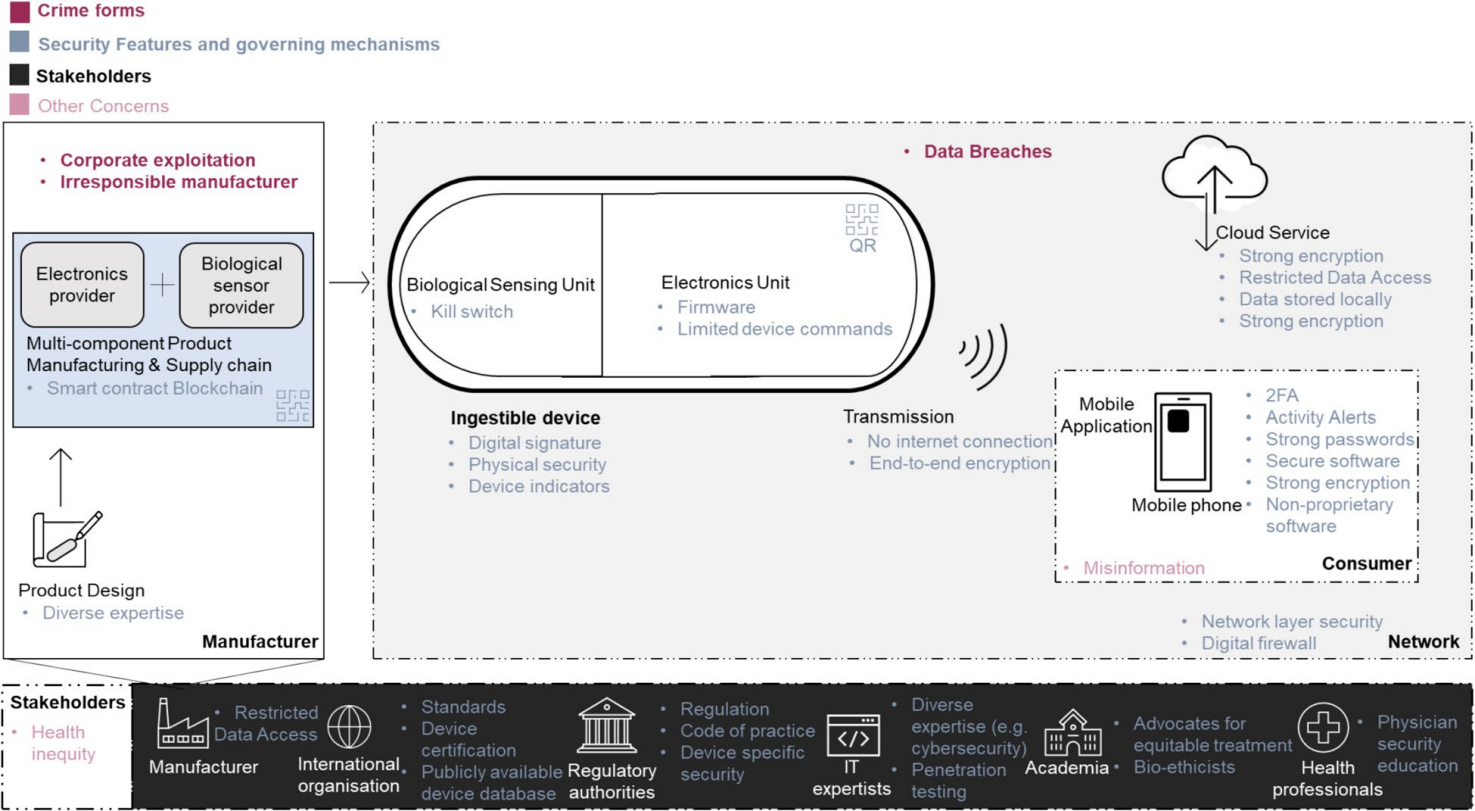
Summary schematic of the Internet of Ingestible Things Landscape defined by the BAKE Framework. Consensus responses from the four expert groups are depicted to contain the Stakeholders (bottom panel) the ingestible device and the network it is connected to (right grey panel). The left panel is specific to the manufacturer of the ingestible device. The ingestible device collects inputs through the biological sensing unit and transmits that through the encapsulated electronics component to a mobile application on a handheld device. Data is then stored on a cloud service for when the consumer of the device wants access. Superimposed on this schematic are the crime forms (red), security features (light blue) and any other concerns (pink) the experts identified during the Delphi study

The secure by design principles for which consensus was reached (70% or more agreed) were end-to-end encryption, firmware, restricted access, strong passwords and standards (Figs. 3 and 4). According to the 73% of experts that strongly endorsed the need for standards, specific guidance for ingestible technology should be developed. Fortunately, organizations such as the US Food and Drug Administration (FDA), the International Organization for Standardization (ISO) and EU Medical Device Coordination Group (MDCG) are already elaborating general standards for medical devices. Albeit not specific to ingestible technology, available guidance can be applied and is discussed here—a summary is in Table 4. FDA and the new EU guidance on cybersecurity are compared to the results of this study, although this is an overview rather than an inclusive and comprehensive analysis. Granlund et al. (2021) MDCG and the newer standard International Electrotechnical Commission (IEC) 80001-5-1:2021 to be comparable. UK latest guidance for example, from the British Standards Institution (BSI) dates back to 2017 (UK/BSI/1014/ST/0217/EN/HL) currently updating medical device regulation in light of exit from the EU, but cites guidance including ISO 14971 risk management, IEC TR 80001-2-8:2016). Considering how the security features of this study relate to such regulatory (e.g., FDA) guidance and best practice for embedded system development and product security, all are in line with (but limited to) medical device cybersecurity management^10^ (FDA-2018-D-3443). For example, on securing data by encryption at rest and in transit, suggested by all four expert groups (Fig. 2 #105, #131), the FDA guidance specifies cryptographic methods^11^ for authentication (Section V.A.1.(b)(iii), Line 414) and per device via unique secure communication key (Section V.A.2.(b)(v), Line 467). The new EU guidance (EU MDCG 2019-16) also states “*control and security of network traffic *via* appropriate measures*” via data encryption (Sect. "Results", Sect. "Secure by design principles from BAKE", p 22).

**Table 4:** Correspondence between defense-in-depth practices from this study and EU and FDA guidance

	This study	FDA-2018-D-3443	EU MDCG 2019–16
1	End-to-end encryption	Section V.A.1.(b)(iii), Line 414; (Section V.A.2.(b)(v), Line 467	Sect. "Results", Sect. "Crime forms revealed by BAKE", pg 15; Sect. "Methods". Section 2.4, pg 11 also Sect. "Results", Sect. "Secure by design principles from BAKE", pg 22
2	Up-to-date firmware and software	Section V.A.1.(b)(v), Line 421, Section V.A.1.(b)(ii), Line 411	Sect. "Methods". Section 2.4, pg 11 also Sect. "Results", Sect. "Crime forms revealed by BAKE", pg 16
3	Restricted data access and protection against unauthorized access	Section V.A.1(a), Line 385; Health Information Portability and Accountability Act (HIPAA) for protection	Sect. "Methods". Sect. "BAKE participants, recruitment and selection", pg 8
4	Strong passwords	Section V.A.1.(a)(v), Line 398)	Sect. "Results". Sect. "Secure by design principles from BAKE", pg 22
5	Penetration testing	Section VII.B.4.(g), Line 732; Section V.B.2.(b), Line 532; Section V.B.2.(c), Line 534; Section V.B.2.(d), Line 536	Sect. "Results". Sect. "Consumer communication areas identified by BAKE", pg 22 and Sect. "Stakeholders defined by BAKE" pg 23
6	Mandating Diverse expertise in design lifecycle)	NA in Section V.B.1.(d), Line 514	NA in Sect. "Results". Sect. "Crime forms revealed by BAKE", pg 15
7	Device specific security standards	NA	NA
8	Open source (non-proprietary)	NA	Doesn’t require open source, but security checking for any open source code: Sect. "Results". Sect. "Consumer communication areas identified by BAKE", pg 23
9	Device certification (e.g., using blockchain technology)	NA	Regulation (EU) 2019/881—ENISA (the European Union Agency for Cybersecurity) cybersecurity certification (EU) No 526/2013 (Cybersecurity Act)^a^
10	External Connection authentication	Section V.A.1.(b)(iv), Line 417	Sect. "Results". Sect. "Corporate espionage and exploitation", pg 18
11	Consumer Security Communication (e.g., labelling)	Section VI., Line 577	NA, Section for information/training to healthcare providers, Sect. "Discussion". Sect. "Specific standards and guidance for ingestible technology", pg 27
12	Security Vulnerabilities disclosure	Section IV. A Cybersecurity Bill of Materials (CBOM), Line 274	Sect. "Methods". Sect. "BAKE participants, recruitment and selection", pg 8

Traditional, non-traditional experts and patients strongly agreed within their groups that data access should be restricted (Fig. 2 #139, #88, #120) to prevent unauthorized access to devices, in agreement with EU guidance (Sect. "Methods". Sect. "BAKE participants, recruitment and selection", p 8). FDA guidance (Section V.A.1(a), Line 385) specifies restricted access but maintaining protection is out of the scope of the document and filled through the Health Information Portability and Accountability Act (HIPAA). 71% of experts in this study strongly agreed with the need for strong password protection, also mentioned in FDA guidance (Section V.A.1.(a)(v), Line 398) specified as not using “hardcoded, default, easily-guessed, easily compromised” credentials and EU guidance (Sect. "Results". Sect. "Secure by design principles from BAKE", pg 22), current Table 4, Row 1. Finally, the need to update firmware and software (for which there was 76% agreement) is in accordance to EU (Sects. 2.4 and "Crime forms revealed by BAKE") and FDA guidance (Section V.A.1.(b)(v), Line 421, Section V.A.1.(b)(ii), Line 411), Table 4, Row 2. According to non-traditional experts, the firmware used in devices should be open to those that use it so that it can be interrogated (Fig. 2 #136, #274. Current requirements include documentation of firmware and software updates (e.g., FDA’s guidance, Section VII. A. 4, Line 685) but does not specify that it needs to be made public. Additional standards from the FDA guidance (for example) includes the Cybersecurity Bill of Materials (CBOM) (capabilities that impact cyber safety and other practices that the manufacturer has to clearly enlist). This is in agreement with the traditional experts who strongly agreed within their group that the devices’ list of commands should be clearly documented (Fig. 2 #121). Moreover, Non-traditional experts strongly agreed within their group that the system surrounding the ingestible device is always at risk, and therefore a mechanism for disclosing vulnerabilities should also be established to enable issues to be logged in a timely fashion (Fig. 2 #74). This is in line with the EU guidance that requires “*instructions for users on how to respond upon detection of a cybersecurity vulnerability or incident*” (Sect. "Methods". Sect. "BAKE participants, recruitment and selection", pg 8), Table 4, Row 12. This is also in line with the Department for Digital, Culture, Media and Sport (DCMS) code of practice. Albeit that the focus of the BAKE was on pre-market assessment, specifically the design phase, other standards such as post-market cybersecurity activities of fielded products were also specified by the Hackathon participants. For example, annual checks, specifically for the updated security of ingestible devices, should be enforced to ensure that manufacturers are keeping up with standards (Fig. 2 #180).

### Specific standards and guidance for ingestible technology

Notwithstanding the availability of guidance such as FDA’s and EU’s described above, documents are fragmented and often behind paywalls. For example newer guidance IEC 80001-5-1:2021 not covered in the comparative overview above is inaccessible unless purchased—unlike FDA’s and EU’s. This introduces an inherent uncertainty and difficulty for manufacturers and although there are continuous efforts in harmonising standards (e.g., EU with new Medical Device Regulation) commercialised medical devices, such as diabetes devices (Sackner-Bernstein, J., 2017), continue to have subpar security solutions. Moreover, although documentation is required as part of the regulatory process detailing technical and security features of manufacturer’s product and systems, these documents are not public—something that participants of BAKE specifically highlighted (e.g., Fig. 2, #310, 346). Additionally, experts in this study all agreed that the design phase of the product life cycle requires more attention. For example, Non-traditional experts strongly agreed within their group that a diverse set of expertise is needed earlier in the product development life cycle of ingestible devices, including bio-ethicists, privacy, and encryption experts as well as advocates for equitable treatment, Fig. 2, #135, #272, #273. Although Secure by design principles and lifecycle requirements exist in current guidance, mandating diverse expertise during the design phase is not specified—as suggested by experts in this study. Moreover, Non-traditional experts specified open sourcing (#207) and decentralizing ingestible technology to which 64.4% experts strongly agreed to (Figs. 3 and 4). For example, hackathon participants agreed that penetration tests should be conducted (66.7%) by manufacturers (or outsourced) and that the results of those tests published (Sackner-Bernstein, J., 2017) (Figs. 3 and 4). Hackathon participants also strongly agreed within their group that blockchain technology could be used (#106) for authenticity and transparency purposes (Mohanty et al., 2020; Paliokas, et al., 2019), Table 4. Overall, having gone through the BAKE Framework, hackathon participants and experts participating in this study generated additional considerations for current regulatory requirements. Interestingly, there was consensus across all four expert groups that the security that the device has should be communicated to consumers of the ingestible technology (Fig. 2, #346, 302, 293). Although not common practice, some progress towards this has been seen in the UK with consumer IoT devices and the newly introduced security label mandate.^12^ X, Author, & X (2020) showed that currently the consumer IoT industry has created “information asymmetries” between manufacturers and consumers due to inaccessible information on the products’ security—making it hard for consumers to assess the security of the products and representing a market failure (Author et al. 2020; X, Author & X, 2020). Moreover, the authors showed that participants of the study were significantly more likely to select a device that carried a label than one that did not and that were willing to pay for the security (Author et al. 2020). Currently it is unclear whether ingestible devices should only be regulated as medical devices or commercial electronics. However, if they do move into consumer-focused products a collaborative and comprehensive risk mapping between MHRA and the DCMS is advised. This may involve the ingestible technology be regulated by additional legislation, as the upcoming security label as described above.

### Exploits within the cyber-physical system

The main challenge in smart environments with interconnected medical devices is the incorporation of biological systems. The three crime forms identified by hackathon participants and non-traditional experts were corporate exploitation, data breaches/system exploits and insecure devices by manufacturers. In addition to the traditional “cyber” exploits, vulnerabilities within cyber-physical systems (CPS) are critical (Geismann, Gerking and Bodden, 2018; Peisert et al., 2014). For example, the ingestible technology can comprise of active biological sensing that, instead of traditional electronic sensing, uses synthetic biology techniques to turn a living biological organism (e.g., bacteria) into a biosensor (that produces light when a certain analyte is present, for example) (Mimee et al., 2018). This information exchange to coordinate a physical interaction—bio-digital technology (Peters et al., 2020)—is difficult to secure as biological systems are not tamper proof (Mueller, 2021) and can lead to three critical areas of attack (sabotage, corporate espionage, and crime/extortion) as identified by experts of this study and as supported by other researcher such as Guttieres et al. (2019). Experts of this study acknowledged these risks and specified the necessity for a “Kill Switch”—an auto-shut down system that could be implemented and initiated when there are any external attempts of modification are detected or if a patient wants to stop transmitting data (Fig. 3 #10, Fig. 2 #241, 113, 128, 91). Kill switches are a common term used in synthetic biology and refer to the induced lethality mechanisms that are engineered as genetic safeguards within engineered biological organisms (Moe-Behrens et al., 2013). This highly technical feature, generated by the hackathon and non-traditional experts, is testament to the inclusion of a diverse set of participants in this study.

### Governance via an independent body with a wider ecosystem of diverse and non-traditional experts

The BAKE Framework supported the identification of governance mechanisms for the unique cyberbiosecurity challenges, including an independent body with a wider ecosystem of diverse and non-traditional experts. The four governance mechanisms suggested by respondents in this study were regulation, standards/code of practice, transparency/education, and an independent governance body. In considering security education, security training for professionals in the medical sciences is limited or non-existent, which no doubt explains why the research and healthcare industries are increasingly vulnerable to attacks in their cyber-physical infrastructures (Kruse et al., 2017; Millett et al., 2019a, 2019b; Mueller, 2021). An independent body or cyber-biosecurity centre (Author 2021), e.g., NIST or similar, could be useful in analysing the ingestible (or related) technology against a security framework. This could allow for example, the assessment of the technology to check if it is made in a hostile state. However, there are additional challenges in addressing this need. For example, the unique challenges of cyber-biosecurity and gap in expertise (Mueller, 2021). In addition to the more intuitive health professionals and regulatory authorities, experts in this study strongly agreed that stakeholders of the ingestible technology should include a wider ecosystem that involves information technology experts but also social science academics such as bio-ethicists.

### BAKE framework is technology-agnostic

A major challenge in developing solutions to the issues discussed is the difficulty of identifying novel risks and threats, including hypothetical vulnerabilities, particularly given that there is no comprehensive framework to effectively capture them (Mueller, 2021). BAKE offers one solution to this. BAKE is technology-agnostic and inclusive of a diverse set of participant experts in identifying novel risks and threats within cyberbiosecurity. This approach has proven effective in generating potential scenarios with the most scenarios generated coming from the hackathon participants, Table 2, and the Non-traditional experts, Table 3—consistent with findings of the 2022 Delphi study conducted (Author et al. 2022). While it cannot demonstrate that it produces more or more useful information, Delphi studies have high levels of attrition, the invited hackathon participants in this study however all completed the Delphi, indicating their commitment.

Engagement with the non-traditional group remains a challenge as indicated by the low number of participants. All non-traditional experts were from the US, with different time zones to the UK (where the study was run), which may have lowered participation, Table 3. Another possible reasoning behind this low participation could be the specific technology selected as a testbed. Implantables are more common to see in this hidden population (Author and X 2021) than ingestible technology (Yetisen, 2018).^13^ Moreover, perhaps if all participants had been recruited to attend the hackathon itself, there may have been a higher participation rate (Meyer, 2020). Fortunately, the BAKE Framework can be implemented for any technology of interest. To the extent that this model was tested on the hardware (the ingestible device), the BAKE Framework can also be used to further investigate the “wetware” of the technology to begin to explore the cyberbiosecurity challenges described in this section. In a post-covid world with more medical devices and processes containing integrated sensors, a huge amount of patient specific data will be harnessed (Mandsberg et al., 2020). BAKE offers a framework for the ongoing assessment and early identification of threats that may help to prevent a crime harvest from occurring in the future. Further assessment on cost effectiveness for ongoing testing and potential payers for this will need to be investigated.

### Limitations, strengths and future work

One of the main strengths of this study was the elicitation of scenarios and the synthesis of opinions from a diverse set of experts to reach a consensus on areas in need of focus ahead of time. The study scope was limited to the identification of risks and potential responses to those risks. To extend this study, the mitigation landscape would be significant to assess in the case that these were compromised.

To the extent that the participants may not be representative that could lead to a focus on some issues and not others, this study was designed specifically to include the four different parallel groups to try and ensure diversity within them.

## Conclusion

The study reported here provides evidence for the utility of the BAKE as a mechanism to identify and act on, at an early stage of design/development, criminally-exploitable vulnerabilities in electronic devices, especially medical products/services using ingestible devices as a case study.

In so doing, BAKE answered key questions regarding the Internet-of-Ingestible-Things, including what areas might be misused, identifying three crime forms. Moreover, five security features and s ecure by design principles were identified by experts to help guide future regulation for the ingestible devices, and four governance mechanisms were suggested. BAKE also addressed who the stakeholders of these devices should be, identifying four stakeholders from multiple disciplines and expert fields. We provide evidence for the utility of BAKE as a forward-thinking mechanism to consider security and crime implications in the design of medical devices ahead of their widespread use during the early design phase of a product lifecycle, and while prototyping.

BAKE was proposed to help prepare for the unique and evolving paradigm of cyber-biosecurity threats catalysed by modern connected devices within the bio and life sciences. With a wider ecosystem of diverse and non-traditional experts, novel risks and threats to cyber-biosecurity can be identified and acted on in good time to reduce the likelihood of an unintended crime harvest occurring in the future.

## Data Availability

Data is included as supplementary information.

## Footnotes

1 Cisco Annual Internet Report (2018–2023) https://www.cisco.com/c/en/us/solutions/collateral/executive-perspectives/annual-internet-report/white-paper-c11-741490.html

2 The term “BAKE” is derived from hyBrid hAcKathon dElphi, and was selected to capture the "baking" process of technology, where each step requires iterative security measures and careful nurturing.

3 We note that although traditional experts did not reach consensus they had 60% agreement within their group that expected crime forms for the ingestible devices may include unconsented data access (Supplementary Table 1 #52), price gouging (Supplementary Table 1 #47) and covert health surveillance (#43) from corporates (Fig. 2). Additionally, 60% of traditional experts strongly agreed within their group that there would be opportunities to counterfeit ingestible devices, (Supplementary Table 1 #45).

4 We note that although the patient group did not reach consensus they had strong agreement within their group that scored 67% for hacking (Supplementary Table 1 #7) and 67% agreement within their group that the ingestible devices will be a target of theft (Supplementary Table 1 #4).

5 We note that although the traditional expert group did not reach consensus they had strong agreement (60%) within their group that there are far less intrusive means of obtaining health data (Fig. 2 #50). An example according to the traditional experts, is the hacking of a medical database that would be much easier to deploy than an attack directed to an ingestible device, if the device was internet-connected.

6 More than 50%.

7 We note that although hackathon participants did not reach consensus, 66.67% agreed within their group that employing cybersecurity specialists could be a legal requirement for an ingestible device provider/manufacturer (Fig. 2 #24).

8 We note that although hackathon participants did not reach consensus, 66.7% agreed within their group with the above (Fig. 2 #241). Additionally, 66.7% of hackathon participants agreed within their group that government agencies (Fig. 2 #240) and international organisations (Fig. 2 #23) could also play a part in regulating ingestible technology. For example, the World Health Organisation could set international standards for manufacture of ingestible technology.

9 We note that a although traditional experts did not reach consensus, 60% strongly agreed within their group that an assessment of potential “uncommon” patient/user physiology should be considered for ingestible technology; for example, device encounters something unusual in a patient’s physiology and decides to act (Fig. 2 #359).

10 Premarket Submissions for Management of Cybersecurity in Medical Devices https://www.fda.gov/regulatory-information/search-fda-guidance-documents/content-premarket-submissions-management-cybersecurity-medical-devices

11 NIST recommended standards for cryptography (e.g., FIPS 140-2, NIST26 Suite B27), or equivalent-strength cryptographic protection for communications channels. (Section V.A.2.(b)(iv), Line 464).

12 Government response to the Regulatory proposals for consumer Internet of Things (IoT) security consultation https://www.gov.uk/government/consultations/consultation-on-regulatory-proposals-on-consumer-iot-security/outcome/government-response-to-the-regulatory-proposals-for-consumer-internet-of-things-iot-security-consultation

13 Although one at-home ingestible device has been reported https://hackaday.io/project/159098-stomach-acid-powered-smart-pill#j-discussions-title and https://hackaday.com/2018/07/07/this-smart-pill-uses-a-stomach-acid-battery/

## References

[CR1] Akartuna, E. A., Johnson, S. D., & Thornton, A. (2022). Preventing the money laundering and terrorist financing risks of emerging technologies: An international policy Delphi study. *Technological Forecasting and Social Change,**179*, Article 121632.

[CR2] Alon, I., Guimón, J., & Urbanos-Garrido, R. (2019). What to expect from assisted reproductive technologies? Experts’ forecasts for the next two decades. *Technological Forecasting and Social Change,**148*, Article 119722.

[CR3] Alsunaydih, F. N., & Yuce, M. R. (2021). Next-generation ingestible devices: Sensing, locomotion and navigation. *Physiological Measurement,**42*(4), Article 04TR01. 10.1088/1361-6579/abedc0 33706294

[CR4] Applegate, S. D. (2013). The dawn of kinetic cyber. In *2013 5th international conference on cyber conflict (CYCON 2013)* (pp. 1–15). IEEE.

[CR5] Avella, J. R. (2016). Delphi panels: Research design, procedures, advantages, and challenges. *International Journal of Doctoral Studies,**11*, 305.

[CR6] Belton, I., MacDonald, A., Wright, G., & Hamlin, I. (2019). Improving the practical application of the delphi method in group-based judgment: A six-step prescription for a well-founded and defensible process. *Technological Forecasting and Social Change,**147*, 72–82.

[CR7] Briscoe, G. (2014). Digital innovation: The hackathon phenomenon.

[CR8] Brodwin, E. (2018). *DNA-Testing Company 23andMe Has Signed a $300 Million Deal With a Drug Giant. Business Insider*. (Accessed August, 2020 13). Available online at: https://www.businessinsider.com/dna-testing-delete-your-data-23andme-ancestry-2018-7?r=US&IR=T

[CR70] Buterin, V. (2014). A next-generation smart contract and decentralized application platform. *white paper,**3*(37), 2–1.

[CR9] Cave, D. R., Fleischer, D. E., Leighton, J. A., Faigel, D. O., Heigh, R. I., Sharma, V. K., Gostout, C. J., Rajan, E., Mergener, K., Foley, A., et al. (2008). A multicenter randomized comparison of the Endocapsule and the Pillcam SB. *Gastrointestinal Endoscopy,**68*, 487–494. 18410941 10.1016/j.gie.2007.12.037

[CR10] Cavoukian, A. (2009). Privacy by design: The 7 foundational principles. *Information and Privacy Commissioner of Ontario, Canada,**5*, 12.

[CR11] Chang, A. M., Gardner, G. E., Duffield, C., & Ramis, M. A. (2010). A Delphi study to validate an advanced practice nursing tool. *Journal of Advanced Nursing,**66*(10), 2320–2330. 20626481 10.1111/j.1365-2648.2010.05367.x

[CR12] Chong, K. P., & Woo, B. K. (2021). Emerging wearable technology applications in gastroenterology: A review of the literature. *World Journal of Gastroenterology,**27*(12), 1149. 33828391 10.3748/wjg.v27.i12.1149 PMC8006095

[CR13] Coutorie, L. E. (1995). The future of high-technology crime: A parallel delphi study. *Journal of Criminal Justice,**23*(1), 13–27.

[CR14] Dalkey, N.C. (1968). Predicting the Future (P-3948), RAND Corporation, https://www.rand.org/pubs/papers/P3948.html

[CR15] Dalkey, N., & Helmer, O. (1963). An experimental application of the delphi method to the use of experts. *Management Science,**9*(3), 458–467.

[CR16] DePasse, J. W., Carroll, R., Ippolito, A., Yost, A., Chu, Z., & Olson, K. R. (2014). Less noise, more hacking: How to deploy principles from MIT’s hacking medicine to accelerate health care. *International Journal of Technology Assessment in Health Care,**30*(3), 260–264. 25096225 10.1017/S0266462314000324

[CR17] Eisen, G. M., Eliakim, R., Zaman, A., Schwartz, J., Faigel, D., Rondonotti, E., & DeFranchis, R. (2006). The accuracy of PillCam ESO capsule endoscopy versus conventional upper endoscopy for the diagnosis of esophageal varices: a prospective three-center pilot study. *Endoscopy,**38*(01), 31–35. 16429352 10.1055/s-2005-921189

[CR18] Enders, A. M., Uscinski, J. E., Klofstad, C., & Stoler, J. (2020). The different forms of COVID-19 misinformation and their consequences. *The Harvard Kennedy School Misinformation Review.,**2020*, 1.

[CR19] Fischer, R. G. (1978). The Delphi method: A description, review and criticism. *Journal of Academic Librarianship,**4*(2), 64–70.

[CR20] Franzosa, E. A., Huang, K., Meadow, J. F., Gevers, D., Lemon, K. P., Bohannan, B. J., et al. (2015). Identifying personal microbiomes using metagenomic codes. *Proceedings of the National Academy of Sciences of the United States of America,**112*, E2930–E2938. 10.1073/pnas.1423854112 25964341 10.1073/pnas.1423854112 PMC4460507

[CR21] Geismann, J., Gerking, C., & Bodden, E. (2018). Towards ensuring security by design in cyber-physical systems engineering processes. In *Proceedings of the 2018 International Conference on Software and System Process* (pp. 123–127).

[CR22] Giannarou, L., & Zervas, E. (2014). Using Delphi technique to build consensus in practice. *International Journal of Business Science & Applied Management (IJBSAM),**9*(2), 65–82.

[CR23] Graham, G., & Mehmood, R. (2014). The strategic prototype “crime-sourcing” and the science/science fiction behind it. *Technological Forecasting and Social Change,**84*, 86–92.

[CR24] Granlund, T., Vedenpää, J., Stirbu, V., & Mikkonen, T. (2021, June). On medical device cybersecurity compliance in EU. In *2021 IEEE/ACM 3rd International Workshop on Software Engineering for Healthcare (SEH)* (pp. 20–23). IEEE.

[CR25] Guttieres, D., Stewart, S., Wolfrum, J., & Springs, S. L. (2019). Cyberbiosecurity in advanced manufacturing models. *Frontiers in Bioengineering and Biotechnology,**7*, 210. 10.3389/fbioe.2019.00210 31552236 10.3389/fbioe.2019.00210 PMC6737271

[CR26] Halvari, S. et al. (2019) Conceptualization of hackathon for innovation management. In *Manchester: The International Society for Professional Innovation Management (ISPIM)*.

[CR27] Joyia, G. J., Liaqat, R. M., Farooq, A., & Rehman, S. (2017). Internet of medical things (IoMT): Applications, benefits and future challenges in healthcare domain. *Journal of Communication,**12*(4), 240–247.

[CR28] Kadambi, A. (2021). Achieving fairness in medical devices. *Science,**372*(6537), 30–31. 33795446 10.1126/science.abe9195

[CR29] Keeney, S., Hasson, F., & McKenna, H. (2006). Consulting the oracle: Ten lessons from using the Delphi technique in nursing research. *Journal of Advanced Nursing,**53*(2), 205–212. 16422719 10.1111/j.1365-2648.2006.03716.x

[CR30] Kim, J., Campbell, A. S., de Ávila, B. E. F., & Wang, J. (2019). Wearable biosensors for healthcare monitoring. *Nature Biotechnology,**37*(4), 389–406. 10.1038/s41587-019-0045-y PMC8183422 30804534

[CR31] Kiourti, A., & Nikita, K. S. (2017). A review of in-body biotelemetry devices: Implantables, ingestibles, and injectables. *IEEE Transactions on Biomedical Engineering,**64*(7), 1422–1430. 28212074 10.1109/TBME.2017.2668612

[CR32] Kitchin, R., & Dodge, M. (2019). The (in) security of smart cities: Vulnerabilities, risks, mitigation, and prevention. *Journal of Urban Technology,**26*(2), 47–65.

[CR33] Kozak, M., & Iefremova, O. (2014). Implementation of the delphi technique in finance. *E-Finanse Financial Internet Quarterly,**10*(4), 36–45.

[CR34] Kruse, C. S., Frederick, B., Jacobson, T., & Monticone, D. K. (2017). Cybersecurity in healthcare: A systematic review of modern threats and trends. *Technology and Health Care,**25*(1), 1–10. 10.3233/THC-161263 27689562 10.3233/THC-161263

[CR35] LaGreca, C. D. E., & Boonthum-Denecke, C. (2017). Survey on the Insecurity of the Internet of Things. In *Symposium on Computing at Minority Institutions (ADMI)*.

[CR68] Linstone, H. A., Turoff, M. (Eds.). (1975). The delphi method. Reading,* MA: Addison-Wesley,* (1975), 3–12.

[CR36] Magni, D., Scuotto, V., Pezzi, A., & Del Giudice, M. (2021). Employees’ acceptance of wearable devices: Towards a predictive model. *Technological Forecasting and Social Change,**172*, Article 121022.

[CR37] Mandsberg, N. K., Christfort, J. F., Kamguyan, K., Boisen, A., & Srivastava, S. K. (2020). Orally ingestible medical devices for gut engineering. *Advanced Drug Delivery Reviews,**165*, 142–154. 32416112 10.1016/j.addr.2020.05.004 PMC7255201

[CR38] Maple, C. (2017). Security and privacy in the internet of things. *Journal of Cyber Policy,**2*(2), 155–184.

[CR39] Merfeld, K., Wilhelms, M. P., Henkel, S., & Kreutzer, K. (2019). Carsharing with shared autonomous vehicles: Uncovering drivers, barriers and future developments—a four-stage Delphi study. *Technological Forecasting and Social Change,**144*, 66–81.

[CR40] Meyer, M. (2020). Biohackers tackle the coronavirus. *Public Understanding of Science*.

[CR41] Millett, K., dos Santos, E., & Millett, P. D. (2019a). Cyber-biosecurity risk perceptions in the biotech sector. *Frontiers in Bioengineering and Biotechnology,**7*, 136. 10.3389/fbioe.2019.00136 31275929 10.3389/fbioe.2019.00136 PMC6593240

[CR42] Millett, K., Dos Santos, E., & Millett, P. D. (2019b). Cyber-biosecurity risk perceptions in the biotech sector. *Frontiers in Bioengineering and Biotechnology,**7*, 136. 31275929 10.3389/fbioe.2019.00136 PMC6593240

[CR43] Mimee, M., Nadeau, P., Hayward, A., Carim, S., Flanagan, S., Jerger, L., Collins, J., McDonnell, S., Swartwout, R., Citorik, R. J., Bulović, V., Langer, R., Traverso, G., Chandrakasan, A. P., & Lu, T. K. (2018). An ingestible bacterial-electronic system to monitor gastrointestinal health. *Science,**360*(6391), 915–918. 29798884 10.1126/science.aas9315 PMC6430580

[CR44] Minkkinen, M. (2019). The anatomy of plausible futures in policy processes: Comparing the cases of data protection and comprehensive security. *Technological Forecasting and Social Change,**143*, 172–180.

[CR45] Moe-Behrens, G. H., Davis, R., & Haynes, K. A. (2013). Preparing synthetic biology for the world. *Frontiers in Microbiology,**4*, Article 5. 23355834 10.3389/fmicb.2013.00005 PMC3554958

[CR46] Mohanty, S. P., Yanambaka, V. P., Kougianos, E., & Puthal, D. (2020). Pufchain: A hardware-assisted blockchain for sustainable simultaneous device and data security in the internet of everything (IoE). *IEEE Consumer Electronics Magazine,**9*(2), 8–16.

[CR47] Mueller, S. (2021). Facing the 2020 pandemic: What does cyberbiosecurity want us to know to safeguard the future? *Biosafety and Health,**3*(01), 11–21. 33015604 10.1016/j.bsheal.2020.09.007 PMC7518802

[CR48] Murch, R. S., So, W. K., Buchholz, W. G., Raman, S., & Peccoud, J. (2018). Cyberbiosecurity: an emerging new discipline to help safeguard the bioeconomy. *Frontiers in Bioengineering and Biotechnology,**6*, 39. 29675411 10.3389/fbioe.2018.00039 PMC5895716

[CR71] Obermeyer, Z., Powers, B., Vogeli, C., & Mullainathan, S. (2019). Dissecting racial bias in an algorithm used to manage the health of populations. *Science*, *366*(6464), 447–453. 10.1126/science.aax2342 31649194

[CR49] Omolara, A. E., Alabdulatif, A., Abiodun, O. I., Alawida, M., Alabdulatif, A., & Arshad, H. (2022). The internet of things security: A survey encompassing unexplored areas and new insights. *Computers & Security,**112*, Article 102494.

[CR50] Paliokas, I., Tsoniotis, N., Votis, K., & Tzovaras, D. (2019). A blockchain platform in connected medical-device environments: Trustworthy technology to guard against cyberthreats. *IEEE Consumer Electronics Magazine,**8*(4), 50–55.

[CR75] Peccoud J, Gallegos J E., Murch R., Buchholz W G., and Raman S (2017) Cyberbiosecurity: from naive trust to risk awareness. Trends Biotechnol. 36, 4–7. doi:10.1016/j.tibtech.2017.10.012 10.1016/j.tibtech.2017.10.012 29224719

[CR52] Peisert, S., Margulies, J., Nicol, D. M., Khurana, H., & Sawall, C. (2014). Designed-in security for cyber-physical systems. *IEEE Security & Privacy,**12*(5), 9–12.

[CR53] Peters, M. A., Jandrić, P., & Hayes, S. (2020). Biodigital technologies and the bioeconomy: The global new green deal? *Educational Philosophy and Theory,**53*, 1–12.

[CR54] Pummerer, L., Böhm, R., Lilleholt, L., Winter, K., Zettler, I., & Sassenberg, K. (2022). Conspiracy theories and their societal effects during the COVID-19 pandemic. *Social Psychological and Personality Science,**13*(1), 49–59.

[CR55] Roe, M., Spanaki, K., Ioannou, A., Zamani, E. D., & Giannakis, M. (2022). Drivers and challenges of internet of things diffusion in smart stores: A field exploration. *Technological Forecasting and Social Change,**178*, Article 121593.

[CR56] Rowe, G., & Wright, G. (1996). The impact of task characteristics on the performance of structured group forecasting. *International Journal of Forecasting,**12*, 73–89.

[CR57] Sackner-Bernstein, J. (2017). Design of hack-resistant diabetes devices and disclosure of their cyber safety. *Journal of Diabetes Science and Technology,**11*(2), 198–202. 27837161 10.1177/1932296816678264 PMC5478035

[CR58] Srinivasan, C. R., Rajesh, B., Saikalyan, P., Premsagar, K., & Yadav, E. S. (2019). A review on the different types of Internet of Things (IoT). *Journal of Advanced Research in Dynamical and Control Systems,**11*(1), 154–158.

[CR59] Steiger, C., Abramson, A., Nadeau, P., Chandrakasan, A. P., Langer, R., & Traverso, G. (2019). Ingestible electronics for diagnostics and therapy. *Nature Reviews Materials,**4*(2), 83–98. 10.1038/s41578-018-0070-3 PMC12393167 40895645

[CR60] Stilgoe, J., Owen, R., & Macnaghten, P. (2013). Developing a framework for responsible innovation. *Research Policy,**42*(9), 1568–1580.

[CR61] Thomas, J., & Harden, A. (2008). Methods for the thematic synthesis of qualitative research in systematic reviews. *BMC Medical Research,**8*(1), 45. 10.1186/1471-2288-8-45 PMC2478656 18616818

[CR62] Tucker, J. D., Tang, W., Li, H., Liu, C., Fu, R., Tang, S., Cao, B., Wei, C., & Tangthanasup, T. M. (2018). Crowdsourcing designathon: A new model for multisectoral collaboration. *BMJ Innovations,**4*(2), 46.

[CR63] Turoff, M. (1970). The design of a policy delphi. *Technological Forecasting and Social Change,**2*, 149–171.

[CR64] Velez, S., Neubert, M., & Halkias, D. (2020). Banking finance experts consensus on compliance in US bank holding companies: An e-Delphi study. *Journal of Risk and Financial Management,**13*(2), Article 28.

[CR65] Vogel, C., Zwolinsky, S., Griffiths, C., Hobbs, M., Henderson, E., & Wilkins, E. (2019). A Delphi study to build consensus on the definition and use of big data in obesity research. *International Journal of Obesity,**43*(12), 2573–2586. 30655580 10.1038/s41366-018-0313-9 PMC6892733

[CR69] Wang, J. K., Pamnani, R. D., Capasso, R., & Chang, R. T. (2018). An extended hackathon model for collaborative education in medical innovation. *Journal of medical systems,**42*(12), 239. 10.1007/s10916-018-1098-z 30328518

[CR66] Yaqoob, T., Abbas, H., & Atiquzzaman, M. (2019). Security vulnerabilities, attacks, countermeasures, and regulations of networked medical devices—a review. *IEEE Communications Surveys and TutoriaLs,**21*(4), 3723–3768.

[CR67] Yetisen, A. K. (2018). Biohacking. *Trends in Biotechnology,**36*(8), 744–747. 29550160 10.1016/j.tibtech.2018.02.011

